# Application of ELISA Technique and Human Microsomes in the Search for 11*β*-Hydroxysteroid Dehydrogenase Inhibitors

**DOI:** 10.1155/2019/5747436

**Published:** 2019-05-12

**Authors:** Daria Kupczyk, Renata Studzińska, Rafał Bilski, Alina Woźniak

**Affiliations:** ^1^Department of Medical Biology and Biochemistry, Ludwik Rydygier Collegium Medicum in Bydgoszcz, Nicolaus Copernicus University, Toruń, Poland; ^2^Department of Organic Chemistry, Ludwik Rydygier Collegium Medicum in Bydgoszcz, Nicolaus Copernicus University, Toruń, Poland

## Abstract

The metabolic syndrome is defined by impaired carbohydrate metabolism and lipid disorders and often accompanied by hypertension, all of which will lead to obesity and insulin resistance. Glucocorticoids play a regulatory role in the metabolism of proteins, lipids, and carbohydrates. There is growing evidence for a role of glucocorticoids in the development of the metabolic syndrome. The most important factor that regulates the access of endogenous glucocorticoids to receptors after release of glucocorticoids and their diffusion into the cytoplasm of target cells is the steroid metabolism involving a microsomal enzyme, 11*β*-hydroxysteroid dehydrogenase (11*β*-HSD). The changes in intracellular glucocorticoid metabolism in the pathogenesis of obesity indicate the participation of modulation by 11*β*-HSD1, which may represent a new therapeutic target for the treatment of diseases such as type 2 diabetes, visceral obesity, or atherosclerosis. The aim of our study was to determine the fast and effective method to assess inhibition activity of compounds in relation with 11*β*-hydroxysteroid dehydrogenase. The material for this study was human liver and kidney microsomes. In this study we used ELISA technique using 96-well microplates coated with antibodies which were specific for analyzed enzymes. The method can quickly and efficiently measure the inhibition of both 11*β*-HSD1 and 11*β*-HSD2. This method can be used to search for and determine inhibitors of this enzyme. Cortisone and cortisol were used as the substrates for corresponding enzyme assays. Furthermore, 3-*N*-allyl-2-thiouracil derivatives were used by us for comparison purposes in developing the method, although, due to their structure, those derivatives have not previously been considered as potential inhibitors of 11*β*-HSD1. 3-*N*-Allyl-2-thiouracil derivatives are a group worth considering, because by modifying their structure (e.g., by introducing other substituents into the pyrimidine ring) it will be possible to obtain an increase in the activity of compounds in this regard. In conclusion, this study shows an efficient and fast method of determining inhibition activity of compounds in relation with 11*β*-hydroxysteroid dehydrogenase.

## 1. Introduction

Obesity is classified as a chronic metabolic disease. The frequency of cases of obesity is increasing especially in highly industrialized countries. It has been confirmed by many epidemiological researchers [1, 2]. Obesity is often accompanied by such phenomena as insulin resistance, dyslipidemias, disorders of carbohydrate metabolism, and hypertension. The phenomena listed above are the part of metabolic syndrome definition [3]. Obesity and overweight are the risk factors for the development of type II diabetes, metabolic syndrome, atherosclerosis, and cardiovascular diseases [4–8]. The main causes of the so-called simple obesity include incorrect nutrition, both qualitative and quantitative, as well as lack of physical activity. The genetic factors may also contribute to the development of simple obesity, although to a lesser extent. The causes of secondary obesity (which may occur in the course of central nervous system disorders, endocrinopathies, drug-induced syndrome due to the action of glucocorticosteroids, antidepressants, estrogens, or some neuroleptics) are more complex [9].

The metabolic syndrome mentioned earlier was first described in 1988 by Reaven [10]. It is recognized when carbohydrate metabolism, lipid disorders, and hypertension are coexisting, which leads to obesity and insulin resistance. Therefore, obesity, especially central, is an important element of the metabolic syndrome, because it is the main factor responsible for the development of insulin resistance [11]. In addition, abdominal obesity has the phenomenon of apparent hypercortisolemia associated with excessive sensitivity of peripheral tissues to cortisol, although its blood level remains within the limits of reference values [12]. Hypercortisolemia helps in the development of metabolic syndrome. Glucocorticoids, resulting in an increase in insulin resistance and accumulation of adipose tissue in the abdominal area, worsen the course of the metabolic syndrome [13].

Glucocorticoids belong to the group of steroid hormones secreted by the adrenal cortex. They are produced from cholesterol. Their secretion is controlled by the hypothalamic-pituitary-adrenocortical axis [14]. Corticoliberin and corticotropin are involved in the regulation of their secretion. Glucocorticoids play a regulatory role in the metabolism of proteins, lipids, and carbohydrates [15, 16]. In high concentrations, glucocorticoids may contribute to changes in the properties of biological membranes. Through the process of integration into the cellular and mitochondrial membranes, changes in the function of membrane proteins may occur and thus affect their permeability and enhance the processes of lipid peroxidation [17]. In the case of hypercortisolemia, fat accumulates in the abdominal region, causing the development of the so-called abdominal obesity, which is one of the elements of the metabolic syndrome [18, 19]. The main glucocorticosteroids are cortisol (hydrocortisone) and corticosterone. In humans, the dominant glucocorticosteroid is cortisol. The biological activity of glucocorticoids determines the presence of the hydroxyl group in the C11 position of the steroid molecule and the oxidation of this group leads to the transformation of the compound into an inactive form. Therefore, both cortisol and corticosterone are biologically active steroids, while cortisone and 11-dehydrocorticosterone are inactive forms [20].

The most important factor that regulates the access of endogenous glucocorticoids to receptors, after release of glucocorticoids and their diffusion into the cytoplasm of target cells, is the steroid metabolism involving a microsomal enzyme, 11*β*-hydroxysteroid dehydrogenase (11*β*-HSD) (EC 1.1.1.146) [21]. It is an enzyme that catalyzes the transformation of cortisol and its inactive cortisone metabolite, corticosterone and 11-deoxycorticosterone. The first reaction occurs in humans, the second in rodents. The author of reports on this interconversion in 1953 was Amelung [22].

**Figure 1:**
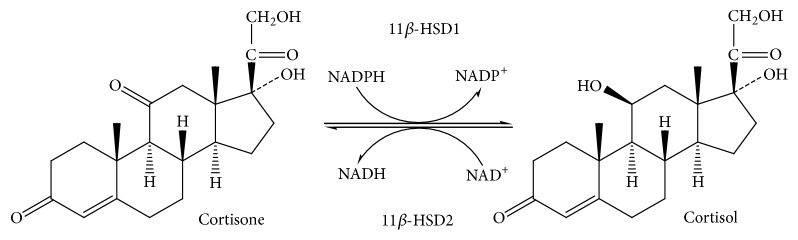
Reactions catalyzed by 11*β*-HSD1 and 11*β*-HSD2.

In humans, two isoforms of this enzyme have been identified, namely, type 1 (11*β*-HSD1) and type 2 (11*β*-HSD2), which are products of two different genes. The first type of enzyme is located on chromosome 1, while the second on chromosome 16 [23, 24]. These isoforms belong to the short-chain dehydrogenase super-family. They differ in terms of affinity for the substrate, direction of the catalyzed reactions, or substrate specificity [20]. They contain an N-terminal sequence that allows them to be anchored in the membrane of the endoplasmic reticulum (ER). The catalytic section of 11*β*-HSD1 is directed towards the ER light, whereas in the case of 11*β*-HSD2 towards the cytoplasm [25]. 11*β*-HSD1 dehydrogenase is found mainly in adipose tissue, liver, and lungs, whereas 11*β*-HSD2 is expressed in tissues associated with mineralocorticoid activity, namely, kidney, placenta, large intestine, and salivary glands. It converts cortisol into metabolically weak cortisone [26, 27], while the role of 11*β*-HSD1 is to convert cortisone to the active form of cortisol (Figure 1).

Inhibition of 11*β*-hydroxysteroid dehydrogenase type 1 activity reduces cortisol levels, which may result in a decrease in fat mass, decrease in glucose levels in blood among patients with type 2 diabetes [28, 29], and lowering of total cholesterol level [30]. Therefore, from the therapeutic point of view, it is important to search new compounds that would be selective 11*β*-HSD1 inhibitors.

Previous reports show beneficial effects of reducing 11*β*-HSD1 activity, which is why attempts are being made to apply its inhibitors in the pharmacotherapy of civilization diseases. The first proposed blocker was arylsulfonamidothiazole, which in a nonselective manner inhibited 11*β*-HSD1. However, in studies conducted with mice, it was found that, despite lowering blood glucose, it did not increase peripheral glucose uptake [31, 32]. Similar results were achieved by the use of a nonspecific 11*β*-HSD1-carbenoxolone inhibitor in humans [33]. Currently, compounds are being sought that would selectively inhibit the activity of the enzyme, and their effect would be to reduce body weight, lower triglycerides, total cholesterol, and insulin, or prevent atherosclerotic plaque formation.

## 2. Materials and Methods

### 2.1. Reagents

The reagents were bought commercially and used without further purification: 18-beta-glycyrrhetinic acid from Acros Organic, phosphate buffer powder, cortisone, NADPH tetrasodium salt from Sigma-Aldrich, dimethylsulfoxide from POCh Poland, carbenoxolone (sodium salt) from Cayman Chemical Company, pooled human liver microsomes, mixed gender (1mL, 20 mg/mL Lot No. 1410013 XenoTech), Cortisol Elisa Ref Dk0001n Lot No. 4297 DiaMetra, ELISA Kit for 11-Beta-Hydroxysteroid Dehydrogenase Type 1 Lot No. L160706125 Cloud-Clone Corp., Human Kidney Microsomes, mixed gender (0.5 mL, 10 mg/mL Lot No. 1610088 XenoTech), Enzyme-Linked Immunosorbent Assay (ELISA) Kit for 11-Beta-Hydroxysteroid Dehydrogenase Type 2 Lot No. L1707221781, Cloud-Clone Corp., PBS Lot No. H161008 Pan Biotech.

### 2.2. Preparation of the Compounds** 1** -** 7** [34–36]

The compounds** 1** -** 6** used in determination of 11*β*-hydroxysteroid dehydrogenase inhibition were synthesized earlier. The compounds were synthesized, respectively:** 1** and** 4** according to [34], the compounds** 2** and** 3** according to [35], and the compounds** 5** and** 6** according to [37]. The compound** 7** (carbenoxolone) was purchased commercially from Cayman Chemical Company.

### 2.3. Determination of 11*β*-HSD1 Activity in Human Liver Microsomal Fractions

The enzyme assay was performed using a microtest plate with 96 wells coated with the 11*β*-hydroxysteroid type 1 dehydrogenase specific antibody. The manufacturer's instruction (Cloud-Clone Corp. SEC268Hu) was used as a guidance to perform the method. As the material for this study, the microsomal fraction of human liver at 20 mg/mL supplied from XenoTech was used. The activity of 11*β* -HSD1 was determined with 4 microsomal concentrations (1 *μ*L/mL, 2.5 *μ*L/mL, 5 *μ*L/mL, and 10 *μ*L/mL) from three independent determinations.

### 2.4. The Reduction Reaction of Cortisone to Cortisol

The reduction reaction of cortisone to cortisol was carried out on 96-well microplate in the presence of the enzyme 11*β*-HSD1 in a total volume of 100 *μ*L: for this purpose, 20 *μ*L of a cortisone/NADPH mixture with a final concentration of 200 nM/2 mM, 10 *μ*L of microsomes (1.13 *μ*g/mL 11*β*-HSD1) solution in PBS to obtain the final quantity of 2.5 *μ*g, 60 *μ*L phosphate buffer pH 7.4, and 10 *μ*L of the appropriate inhibitor solution prepared in a DMSO/water system in a ratio of 1/99 with a final concentration of 10 *μ*M. The microplate prepared in this way was incubated for 150 minutes at 37°C. During the incubation an orbital shaker was used. After the incubation was complete, the reaction was stopped by adding 10 *μ*L of a solution that contained 100 *μ*M of 18*β*-glycyrrhetinic acid in PBS.

### 2.5. Determination of 11*β*-HSD1 Inhibition

The quantity of cortisol formed from cortisone in the reaction described in Section 2.4 was determined using a cortisol ELISA kit DiaMetra. For this purpose, from each well in which the reduction reaction of cortisone was carried out (see Section 2.4), 10 *μ*l of the reaction mixture was taken and placed in a corresponding place on a 96-well microplate. The method was carried out in accordance with the manufacturer's instructions (Cortisol Elisa Ref Dk0001n Lot No. 4297 DiaMetra). The concentration of the obtained cortisol in the analyzed samples was obtained using the standard curve. The percent of 11*β*-HSD1 inhibition was determined in relation to the zero-sample (a sample in which 10 *μ*L of DMSO/H_2_O 1/99 solution was added instead of the inhibitor's solution).

### 2.6. Determination of 11*β*-HSD2 Activity in Human Kidney Microsomal Fractions

The enzyme assay was performed using a microplate with 96 wells coated with the 11*β*-hydroxysteroid type 2 dehydrogenase specific antibody. The method was carried out according to the manufacturer's instructions (Cloud-Clone Corp. SEF183Hu). The microsomal fraction of human kidney at 10 mg/mL supplied from XenoTech was used as the material for the study. The activity of 11*β* –HSD2 was determined with 4 microsomal concentrations (1 *μ*L/mL, 2.5 *μ*L/mL, 5 *μ*L/mL, and 10 *μ*L/mL) from three independent determinations.

### 2.7. The Oxidation Reaction of Cortisol to Cortisone

The oxidation reaction of cortisol to cortisone was carried out on 96-well microplates in the presence of the enzyme 11*β*-HSD2 in a total volume of 100 *μ*L: for this purpose, 20 *μ*L of a cortisol/NAD mixture with a final concentration of 200 nM/2 mM, 10 *μ*L of microsomes (0.39 *μ*g/mL 11*β*-HSD1) solution in PBS to obtain the final quantity of 2.5 *μ*g, 60 *μ*L phosphate buffer pH 7.4, and 10 *μ*L of the appropriate inhibitor solution prepared in a DMSO/water system in a ratio of 1/99 with a final concentration of 10 *μ*M. The microplates prepared in this way were incubated for 150 minutes at 37°C. During the incubation, an orbital shaker was used. After the incubation was complete, the reaction was stopped by adding 10 *μ*L of a solution that contained 100 *μ*M of carbenoxolone in PBS.

### 2.8. Determination of 11*β*-HSD2 Inhibition

The quantity of unreacted cortisol (in the reaction described in Section 2.7) was determined using a cortisol ELISA kit DiaMetra. For this purpose, from each well in which the oxidation reaction of cortisol was performed (see Section 2.7), 10 *μ*l of the reaction mixture was taken and placed in a suitable place on a 96-well plate. The method was performed in accordance with the manufacturer's instructions (Cortisol Elisa Ref Dk0001n Lot No. 4297 DiaMetra). The concentration of the unreacted cortisol in the analyzed samples was obtained using the standard curve. The percent of 11*β*-HSD2 inhibition was determined in relation to the zero-sample (a sample in which 10 *μ*L of DMSO/H_2_O 1/99 solution was added instead of the inhibitor's solution).

## 3. Results and Discussion

Currently conducted research's results show beneficial effects of the reduction of 11*β*-HSD1 activity and, due to this fact, attempts are being made to apply its inhibitors in the pharmacotherapy of civilization diseases. In the search for effective and selective 11*β*-HSD1 inhibitors in recent years, attention has been paid to (1,3-benzothiazol-2-yl) benzenesulfonamides [37], adamantyl amides [38], thiazolo[3,2-*a*]pyrimidine-5-one derivatives [39], and thiazole derivatives (including BVT-14225, BVT-2733, and BVT-3498) [31, 40, 41].

**Figure 2:**
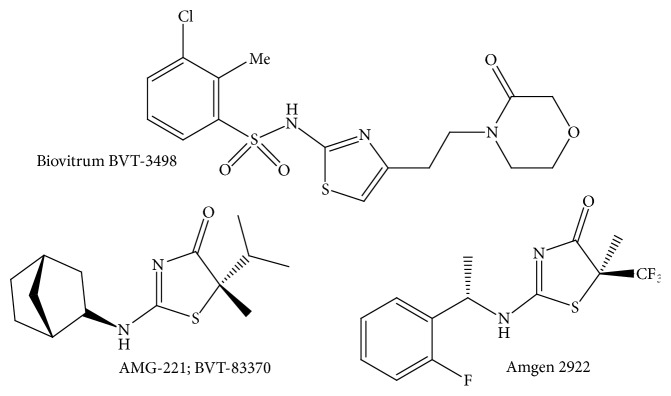
The inhibitors of 11*β*-hydroxysteroid dehydrogenase type 1.

In this group of compounds, Biovitrum BVT-3498 deserves particular attention (Figure 2). In spite of the fact that carbenoxolone also has an inhibitory effect on 11*β*-HSD2, it has undergone clinical trials and successfully completed the second phase of research in 2002. However, in 2005, research for unexplained reasons was suspended [42].

Highly selective 11*β*-HSD1 inhibitors were found to be 2-aminothiazol-4(5*H*)-one derivatives, e.g., Amgen 2922 and AMG-221 (BVT-83370) (Figure 2) [42–45]. AMG-221 (BVT-83370) has successfully passed the phase I of clinical trials. This association has been studied on the obese group and despite good prognosis (IC_50_ = 0.975 ng/mL and maximum extent of enzyme inhibition in adipose tissues of I_max_ = 1.19 ng/mL) in 2011, the study on the implementation of this compound as a drug was cancelled. Therefore, substances that would selectively inhibit 11*β*-hydroxysteroid dehydrogenase type 1 are still being sought.

In order to preestimate the applicability of the compounds as 11*β*-HSD1 inhibitors, it is necessary to develop a rapid, effective, and relatively inexpensive method to determine the inhibition of this enzyme. Previous studies on 11*β*-HSD inhibition were mainly based on scintillation proximity assay (SPA) [40, 43, 46]. The purified enzyme [43] or whole cells (CHO cell line stably overexpressing human or mouse 11*β*–HSD1 [43], Chinese hamster ovary cells stably transfected with human 11b-HSD1 [46], and human embryonic kidney cell cultures (HEK293) cell-based assay [40]) were used to perform the necessary reactions.

We decided to develop a fast, easy, and relatively cheap method of determination of inhibiting both enzyme isoforms in order to be able to carry out the rapid screening of the synthesized compounds in this direction. For this purpose, to avoid the multiplication of cells containing the desired dehydrogenase isoform, as well as the proper preparation of pure enzyme for the reaction, we decided to use commercially available microsomal fractions. Determination of 11*β*-HSD1 in human liver microsomal fractions and 11*β*-HSD2 in human kidney microsomal fractions showed that they contain an amount of enzyme sufficient to affect the conversion of cortisone to cortisol and inversely. The advantage of microsomes is that they do not require time-consuming preparation and can be used immediately after dilution in PBS.

After the reduction reaction of cortisone using human liver microsomal fractions, in order to determine whether test compounds inhibit the activity of 11*β*-HSD1, the amount of obtained cortisol was determined by ELISA using cortisol ELISA kit DiaMetra. It is also a quick technique that allows the determination of cortisol levels in many samples simultaneously.

**Figure 3:**
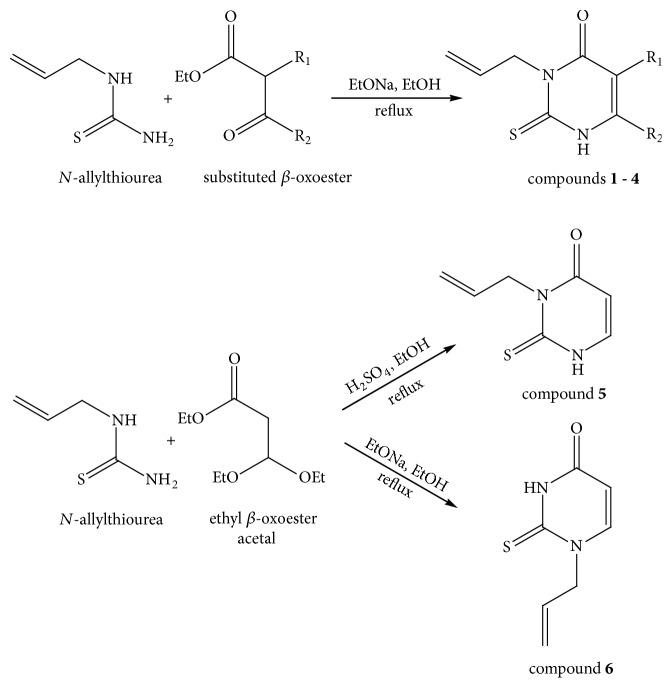
Synthesis of thiouracil derivatives** 1** –** 6**.

In order to verify the correctness of the method developed in our study, we have tested the inhibition of 11*β*-HSD1 by carbenoxolone—a known inhibitor of this enzyme. For comparison, compounds that theoretically should not inhibit 11*β*-HSD1 activity were also used in the study. We focused our attention on* N*-allyl-2-thiouracil derivatives (Table 1). These compounds are obtained by reacting* N*-allylthiourea with the corresponding *β*-oxoesters or their acetals in a basic or acidic medium [34–36] (Figure 3).

Before checking the method, compounds** 1** -** 6** were analyzed using the PASS online program. PASS (Prediction of Activity Spectra for Substances) is a software product designed as a tool for evaluating the general biological potential of an organic drug-like molecule. PASS provides simultaneous predictions of many types of biological activity based on the structure of organic compounds. Thus, it can be used to estimate the biological activity profiles for virtual molecules, prior to their chemical synthesis and biological testing [47]. The use of PASS online software consists in presenting the structural formula of the compound we are interested in. The software compares the structure of a given compound with compounds of known biological activity found in the database. On this basis, it estimates the likelihood of the activity of a given compound in a specific direction. We use the PASS online program to design compounds that would have specific biological activity of interest to us. We also used it in the design of molecules that would show inhibitory activity towards 11*β*-HSD1 [41]. In this way, we received compounds whose activity provided by the PASS online program was confirmed in* in vitro* tests.

There are many possible biological effects of these compounds; however, for none of them, the PASS program does not predict activity towards inhibition of 11*β*-hydroxysteroid dehydrogenase. Unexpectedly, it turned out that these compounds inhibit the enzyme activity to a small extent (from 8.82 to 39.71% at an inhibitor concentration of 10 *μ*M). Such a low percentage of inhibition at such high concentration of inhibitor gives these compounds no practical use as 11*β*-HSD1 inhibitors. However, such large differences in the inhibition of enzyme activity between the known inhibitor, carbenoxolone, and potentially inactive compounds confirm the effectiveness of the method developed by us.

The fact that 3-*N*-allyl-2-thiouracil derivatives, although PASS does not show the probability of inhibiting 11*β*-HSD1, inhibit the activity of this enzyme makes it worth looking at this group of compounds in the search for new 11*β*-HSD1 inhibitors. There is a chance that a slight modification of the structure (e.g., introduction of other substituents to the pyrimidine ring) will increase the activity of compounds.

The effectiveness of our method of determining 11*β*-HSD1 inhibition using human liver microsomal fractions and ELISA technique prompted us to carry out analogous tests to determine the inhibition of the enzyme isoform 2. For this purpose, the human kidney microsomes containing 11*β*-HSD2 were used. In their presence, the cortisol oxidation reaction was carried out. To determine the degree of inhibition of 11*β*-HSD2 we decided to use the same ELISA kit that we used in the case of the reverse reaction. This time we determined the concentration of unreacted cortisol, which allowed us to calculate the concentration of cortisone in postreaction mixtures and consequently to calculate the % inhibition of 11*β*-HSD2 by the inhibitor. Assays for inhibition of 11*β*-HSD2 were conducted for two known inhibitors: carbenoxolone (15.06% at a concentration of 10 *μ*M) and 18*β*-glycyrrhetinic acid (10.96% at a concentration of 10 *μ*M).

## 4. Conclusion

In conclusion, we used ELISA technique using 96-well microplates as a method that can quickly and efficiently measure the inhibition of both 11*β*-HSD1 and 11*β*-HSD2. This method can be used to search for and determine inhibitors of this enzyme. Another advantage of using this technique is the relatively inexpensive cost of the measurement. In the diagnostic and therapeutic process, there is a constant need to provide and improve therapeutic agents. Hence in our study these tests have been undertaken. Furthermore 3-*N*-allyl-2-thiouracil derivatives, although due to their structure have not previously been considered as potential inhibitors of 11*β*-HSD1, are a group worth considering, because by modifying their structure (e.g., by introducing other substituents into the pyrimidine ring) it will be possible to obtain an increase in the activity of compounds in this regard.

## Figures and Tables

**Table 1:** *N*-allyl-2-thiouracil derivatives.

No.		M.p. of **1** – **6** / lit. M.p. (°C)	% of inhibition 11*β*-HSD1 10*μ*M^1^	% of inhibition 11*β*-HSD2 10*μ*M^1^
1.	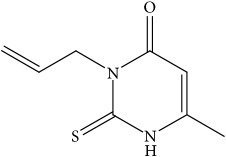	192.5-194 /193.5-194.5 [34]	8.82±0.86	28.08±1.02
2.	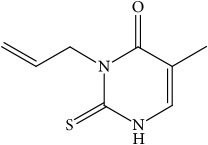	153-154 / 154.5-155 [35]	30.88±1.29	19.18±1.45
3.	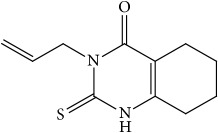	204-205.5 /201-203.5 [35]	35.29±2.01	37.33±2.03
4.	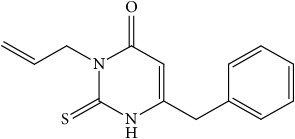	176-177.5 /177.5-179.5 [34]	39.71±1.94	24.66±2.09
5.	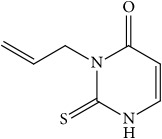	121-122 / 119.5-121 [36]	30.88±1.53	16.78±1.24
6.	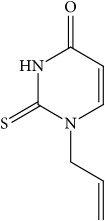	188-189.5 / 189-191 [36]	38.24±1.85	23.29±1.35
7.	carbenoxolone	-	89.70±3.45	15.06±2.06
8.	18*β*-glycyrrhitinic acid	-	88.24±2.05	10.96±3.01

^1^The inhibition of 11*β* -HSD1 and 11*β* –HSD2 was determined with inhibitors concentration 10 *μ*M from three independent determinations.

## Data Availability

The data used to support the findings of this study are included within the article.
